# Supplementary material for: Interactions between Streptococcus agalactiae and Candida albicans affect persistence and virulence
Source: Infect Immun. 2026 Jan 7;94(2):e00528-25. doi: 10.1128/iai.00528-25 (PMC12890033; doi:10.1128/iai.00528-25)
Supplement: Supplemental material — Fig. S1 to S9. [file iai.00528-25-s0001.pdf]

Supplemental Figures

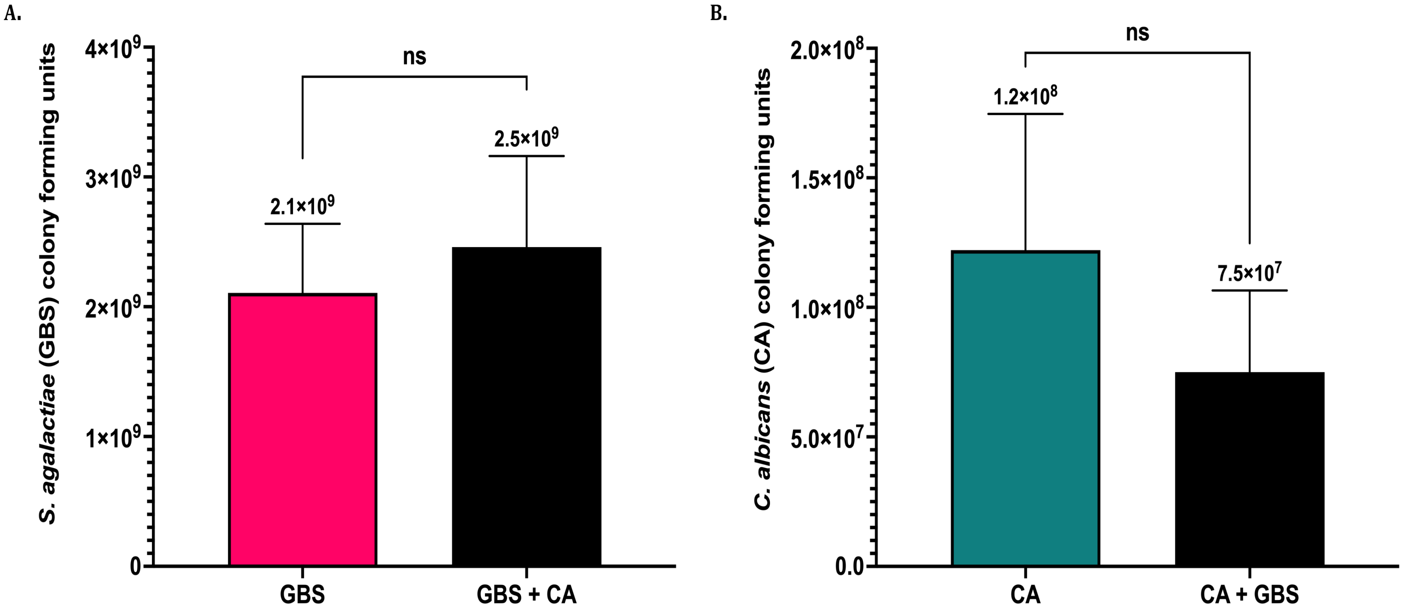

Supplemental Figure 1: GBS and *C. albicans* growth was not altered in co-cultures compared to solo cultures of the pathogens following 24hr culture in nutrient rich (THY) media GBS and C. albicans were incubated in nutrient rich media in solo and in co-cultures and viable colony counts were calculated following 24 hours of incubation **A.** GBS growth in solo and co-cultures with *C. albicans* following 24 hours of incubation **B.** *C. albicans* growth in solo and co-cultures with GBS following 24 hours of incubation. Experiments were performed in triplicate and statistical significance was calculated using an unpaired two-tailed student's t-test $*p<0.05$.

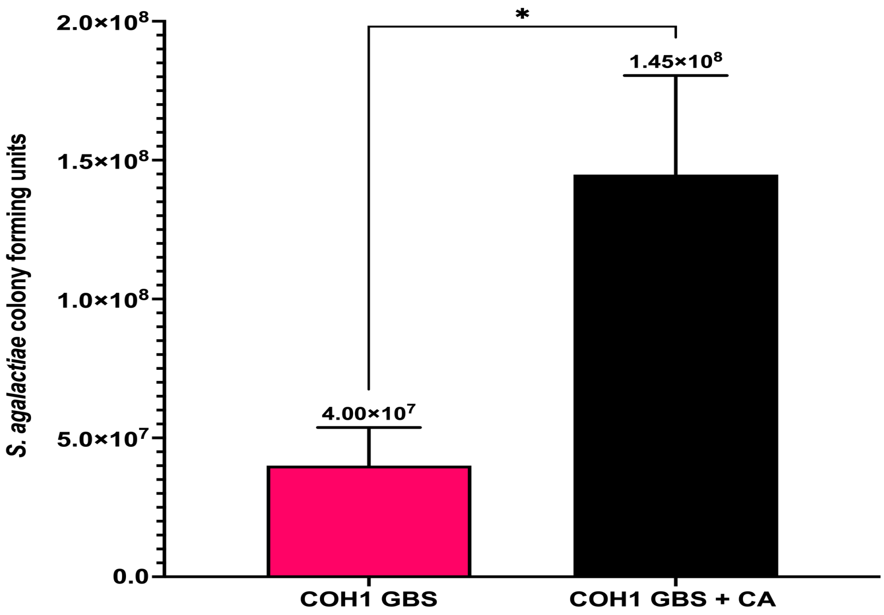

Supplemental Figure 2: GBS increase in growth when co-cultured with *C. albicans* is not serotype dependent. Colony growth of GBS COH1 (serotype III) following solo or co-culture with *C. albicans* at 24 hours post culture in nutrient poor media. Experiment was repeated 4 times and statistical significance was calculated using an unpaired two-tailed student's t-test *$p<0.05$.

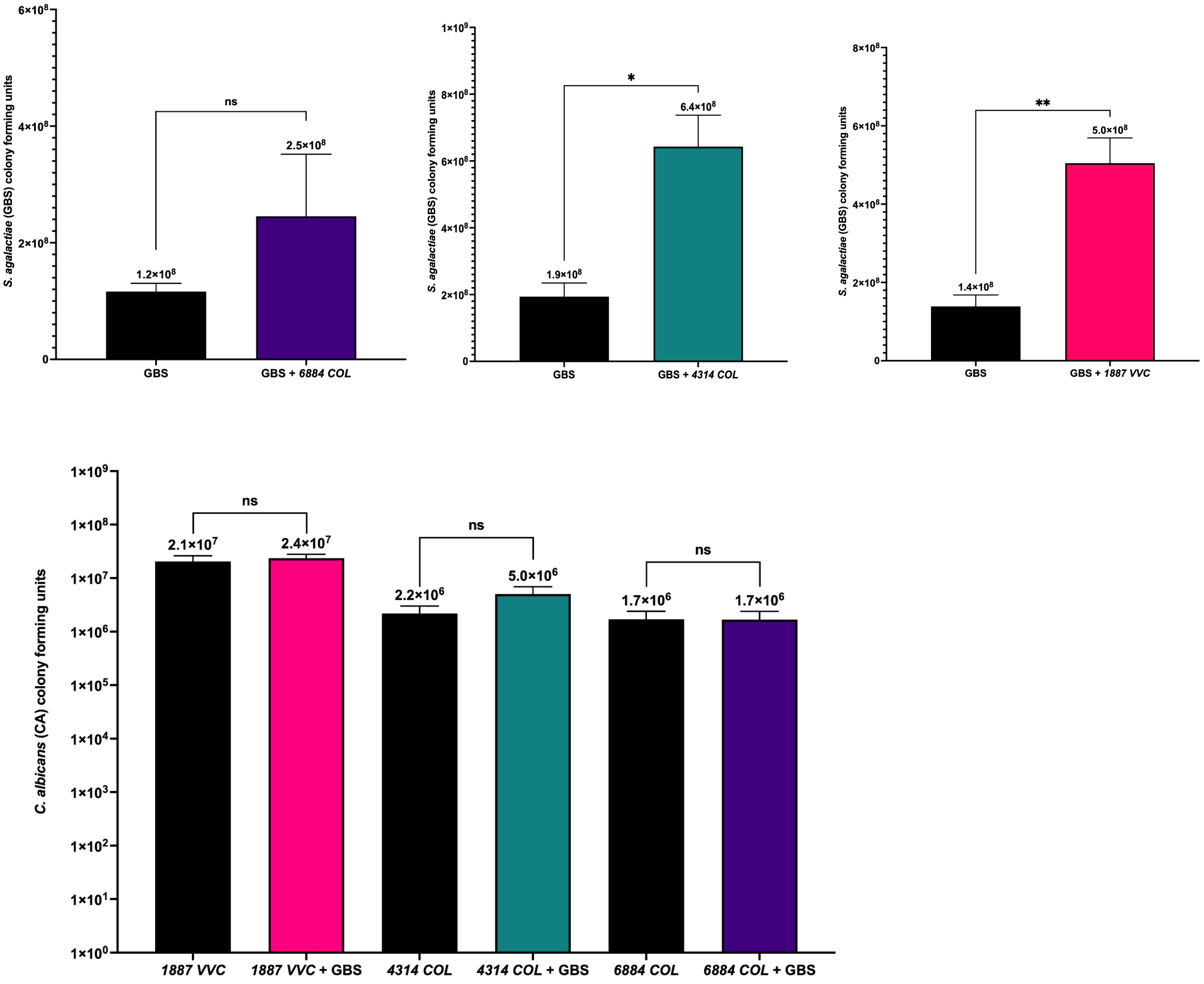

Supplemental Figure 3. The ability of *C. albicans* to enhance the growth of GBS in co-cultures appears to be strain dependent. **A.** Colony growth of GBS following solo or co-culture with three different clinical strains of *C. albicans* at 24 hours in nutrient poor media. **B.** Colony growth of *C. albicans* growth following solo or co-culture with GBS at 24 hours post culture in nutrient poor media. was not significantly different in the presence of GBS for any of the *C. albicans* strains. Results are from combined experiments, and experiments were performed in triplicate. *C. albicans* strains: VVC indicates a strain from a vulvovaginal candidiasis patient, and COL indicates a strain from an asymptomatic individual (46,47). 1887, strain SP-01887; 4314, strain SP-14314; 6884, strain SP-16884. Statistical significance was calculated by unpaired two tailed student t-test, *p<0.05, **p<0.005.

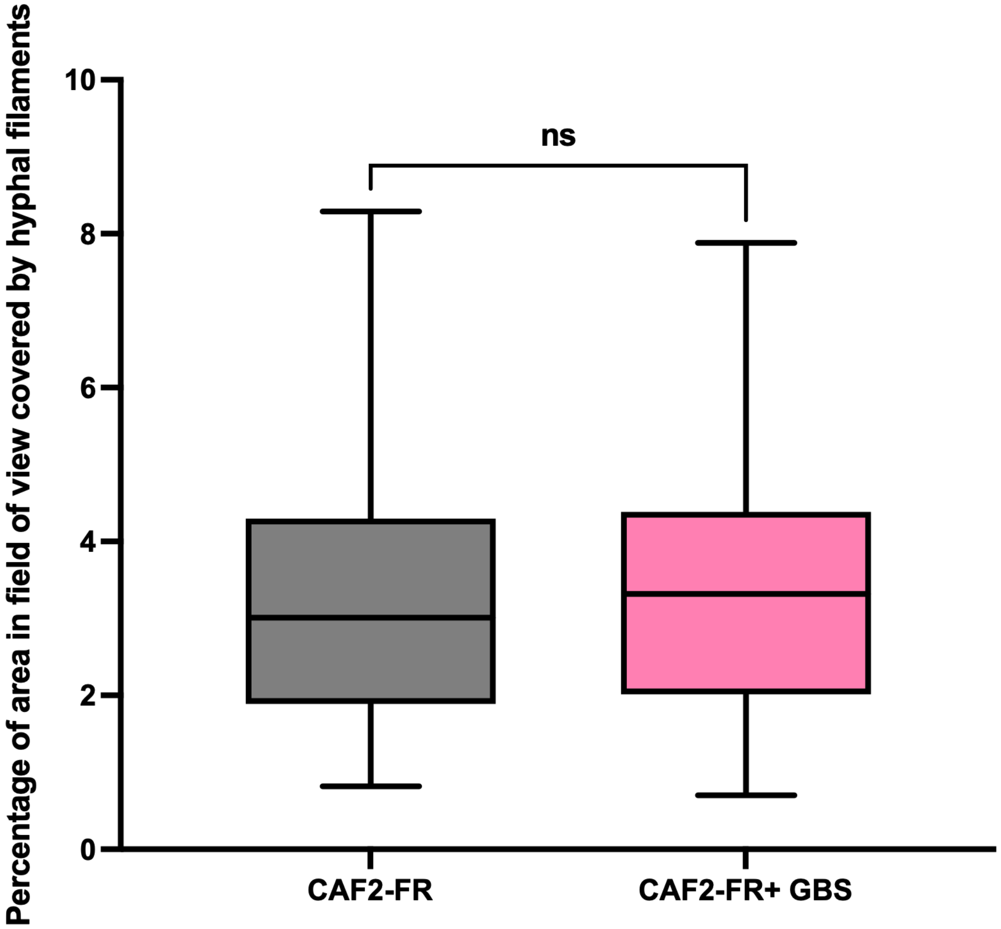

Supplemental Figure 4: GBS strain GBS 515 does not alter the ability of *C. albicans* to form hyphal filaments in RPMI media. The ability of *C. albicans* to form hyphal filaments in solo or co-cultures with GBS strain GBS 515 was quantified by taking images of solo and co-cultures using confocal microscopy. The percentage of area covered by hyphal filaments in the field of view was calculated using ImageJ. Above results are combined experiments from 3 separate replicates, and statistical significance was quantified using an unpaired two-tailed students t-test.

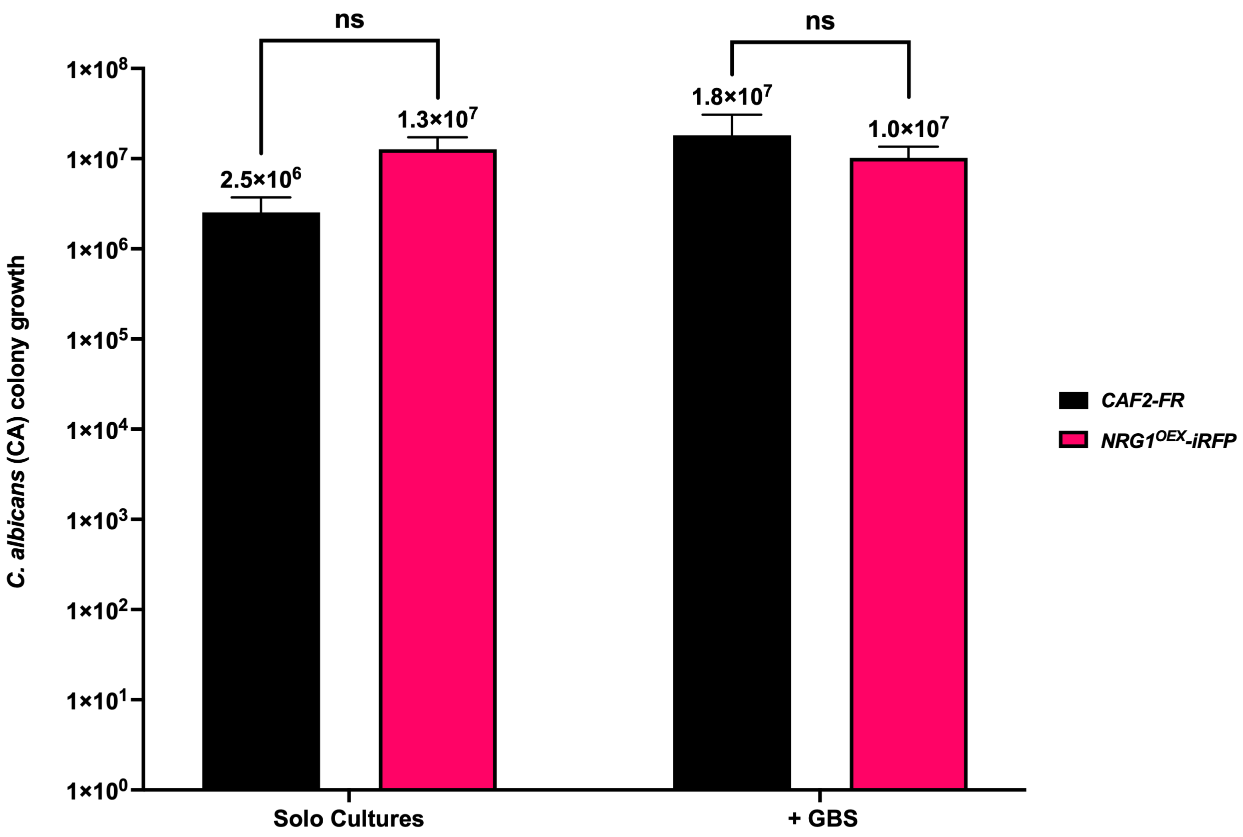

Supplemental Figure 5: The presence of GBS does not significantly change the overall growth of *C. albicans* in nutrient poor media. Growth of *C. albicans* strains CAF2-FR and *NRG1*^{OEX}-iRFP 24 hours post culture in nutrient poor media in solo *C. albicans* cultures or co-cultures with GBS 515. Experiments were performed in triplicate and statistical significance was calculated using unpaired two-tailed student's t-test *$p<0.05$.

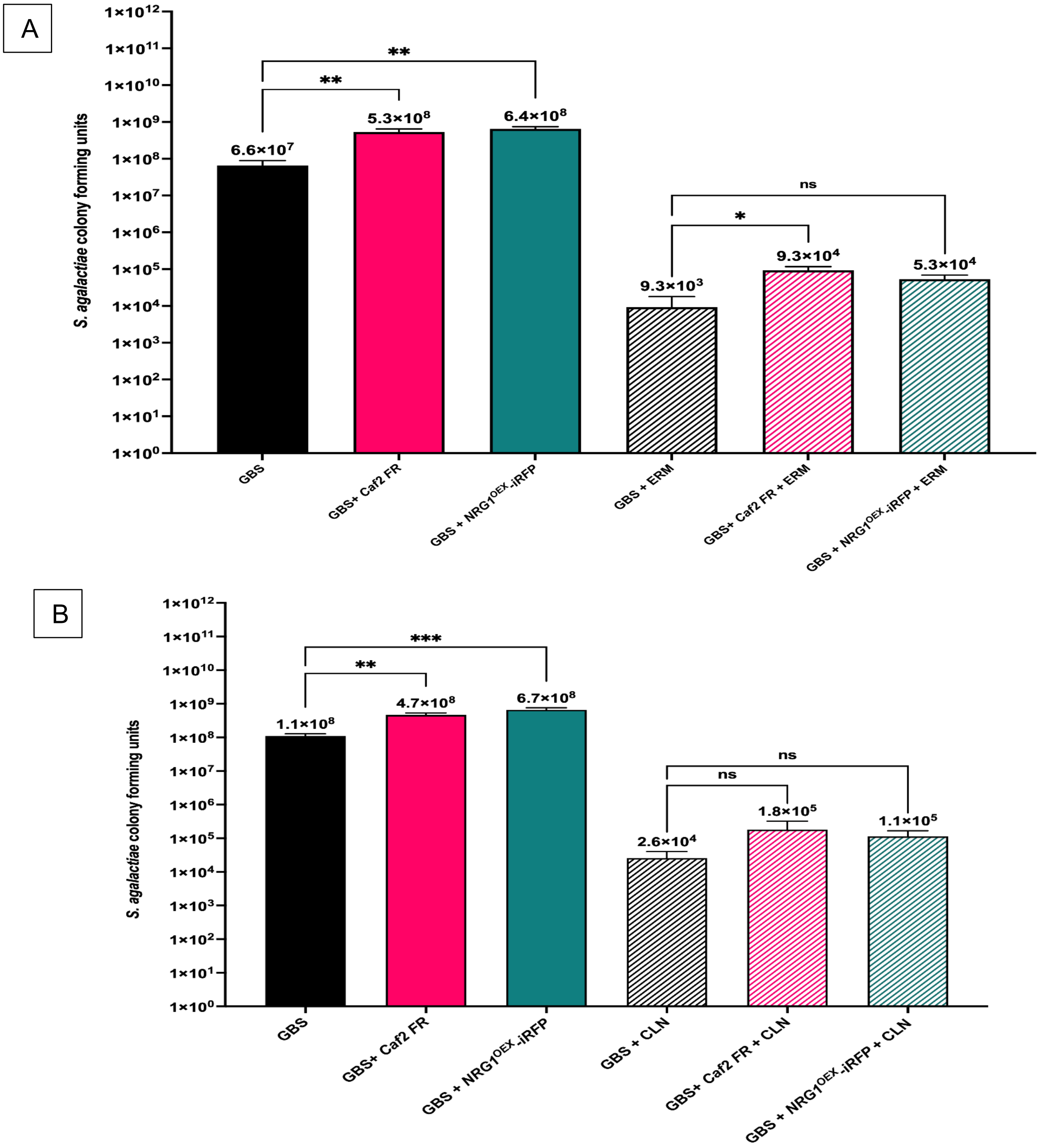

Supplementary Figure 6. Hyphal formation plays a role in antibiotic susceptibility of GBS during co-cultures with *C. albicans.* Both CAF2-FR (WT) and $NRG1^{OEX}$-*iRFP* (yeast-locked) *C. albicans* strains increase growth of GBS during co-cultures. Erythromycin (A) and clindamycin (B) appears to be less effective against GBS when GBS is also cultured with *C. albicans* that is able to form hyphal filaments (CAF2-FR). While the amount of GBS recovered following antibiotic treatment was also enhanced in the presence of Yeast-Locked *C. albicans* ($NRG1^{OEX}$-*iRFP*) it was not as efficient at reducing the efficacy of ERM against GBS as the hyphal *C. albicans* strain, indicating that the presence of hyphal filaments is beneficial to GBS in its ability to resist antibiotic treatment.

A.

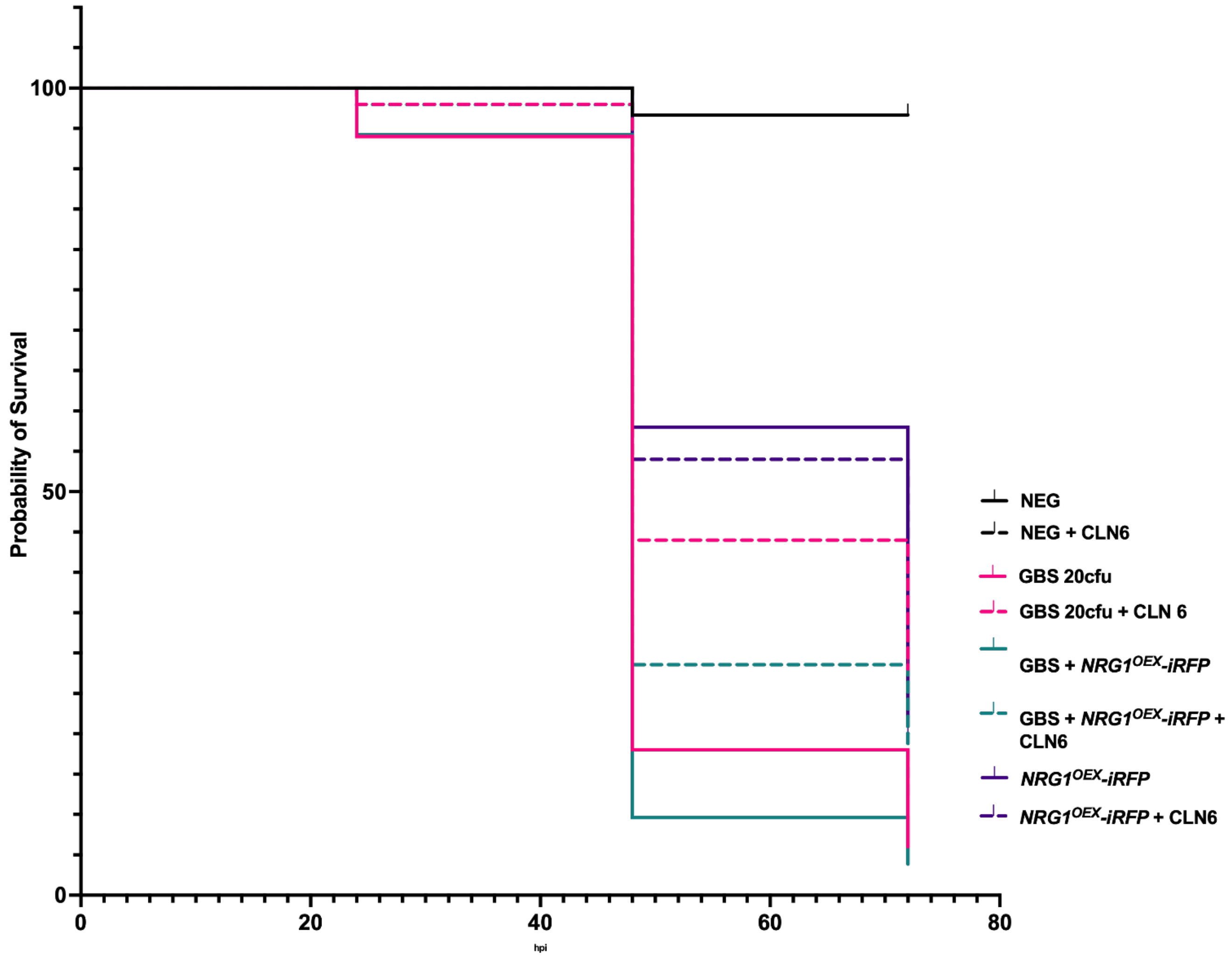

B.

Infection Group	ZF % Survival at 72 hpi
NEG	96.7%
NEG + CLN	96.7%
GBS	6.0%
GBS + CLN6	28.0%
GBS + NRG1^{OEX}-iRFP	3.8%
GBS + NRG1^{OEX}-iRFP + CLN	12.2%
NRG1^{OEX}-iRFP	22.0%
NRG1^{OEX}-iRFP+ CLN	20.0%%

Infection Groups Comparisons	Statistical Significance
GBS vs GBS + CLN	**, p=0.0012
GBS + CLN vs GBS + NRG1^{OEX}-iRFP + CLN	*, p=0.02
GBS + NRG1^{OEX}-iRFP vs GBS + NRG1^{OEX}-iRFP + CLN	*, p=0.02
GBS + NRG1^{OEX}-iRFP vs NRG1^{OEX}-iRFP	****, P<0.0001

Supplemental Figure 7: Hyphal formation is not necessary for protection of GBS from the antibiotic clindamycin by *C. albicans* in yolk-sac infections Solo or co-infections of GBS and *C. albicans* using a yolk sac injection method on 2dpf zebrafish larvae. For solo infections zebrafish were injected with either 20 cfu of GBS 515 or 20 cfu of *C. albicans* yeast-locked strain NRG1^{OEX}-iRFP . For co-infections zebrafish were injected with 10 cfu of GBS and 10 cfu of *C. albicans* (totaling 20 cfu) together. The negative control was 1 nL of 5% PVP in PBS. Experiments were replicated 3 times with 15-20 fish per experimental condition. Graph shows pooled date of 3 experimental replicates **A.** Zebrafish survival of systemic solo and co-infection of GBS and *C. albicans* untreated or treated with 6µg/mL dose of clindamycin in the tank water of the fish at the time of initial infection. **B.** Tables describing zebrafish survival and infection group comparisons where differences in survival percentage were found to be statistically significant for systemic solo and co-infections of GBS and *C. albicans* untreated or treated with 6µg/mL clindamycin Statistical significance of Kaplan-Meier survival curves were calculated using a log rank (Mantel-Cox) test. ****$p<0.0001$.

A.

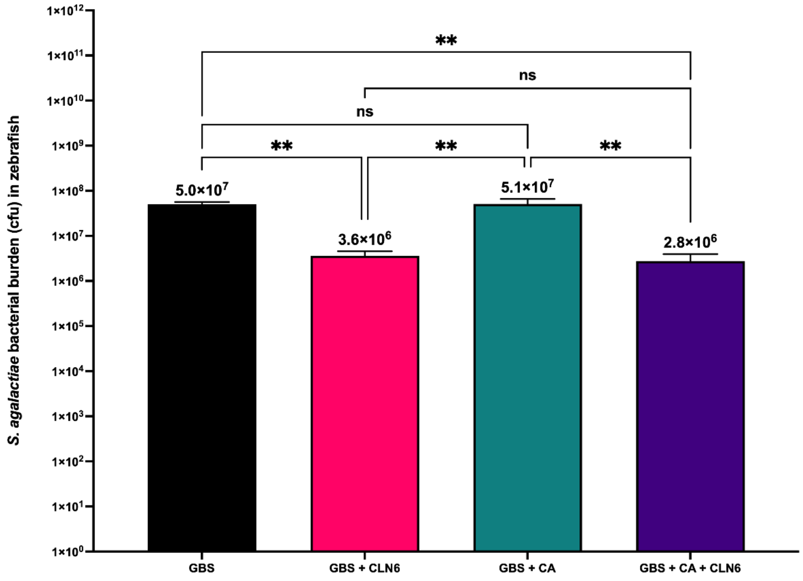

B.

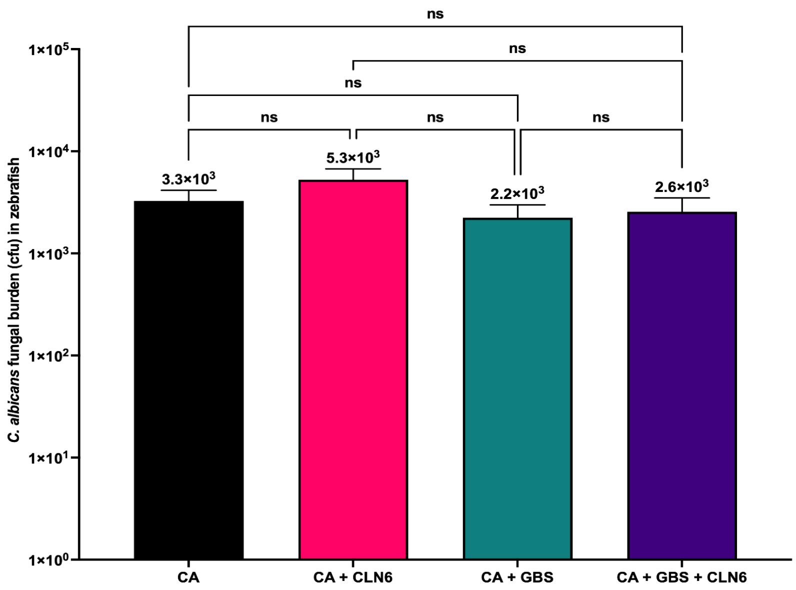

Supplemental Figure 8: **Systemic co-infection with *C. albicans* does not significantly increase GBS burden *in vivo* in untreated infection or infection treated with the antibiotic clindamycin.** The bacterial and fungal burden in infected zebrafish was calculated following yolk sac injection of solo or co-infections of GBS and *C. albicans* into 2dpf larval zebrafish. Solo injections had either 20 cfu of GBS 515 or 20 cfu of *C. albicans.* Co-infections had 10 cfu of GBS and 10 cfu of *C. albicans* (totaling 20 cfu) together. **A.** The average GBS bacterial burden in zebrafish 24 hours following untreated or clindamycin treated systemic solo or co-infection with *C. albicans*. **B.** The average *C. albicans* fungal burden of zebrafish 24 hours following untreated or clindamycin treated solo or co-infection with GBS. Experiments were performed 4 times, with 8 fish homogenized and plated per infection replicate (n=256 fish). Above results are from combined experiments, with statistical significance calculated using one-way ANOVA with Tukey's correction *p<0.005.

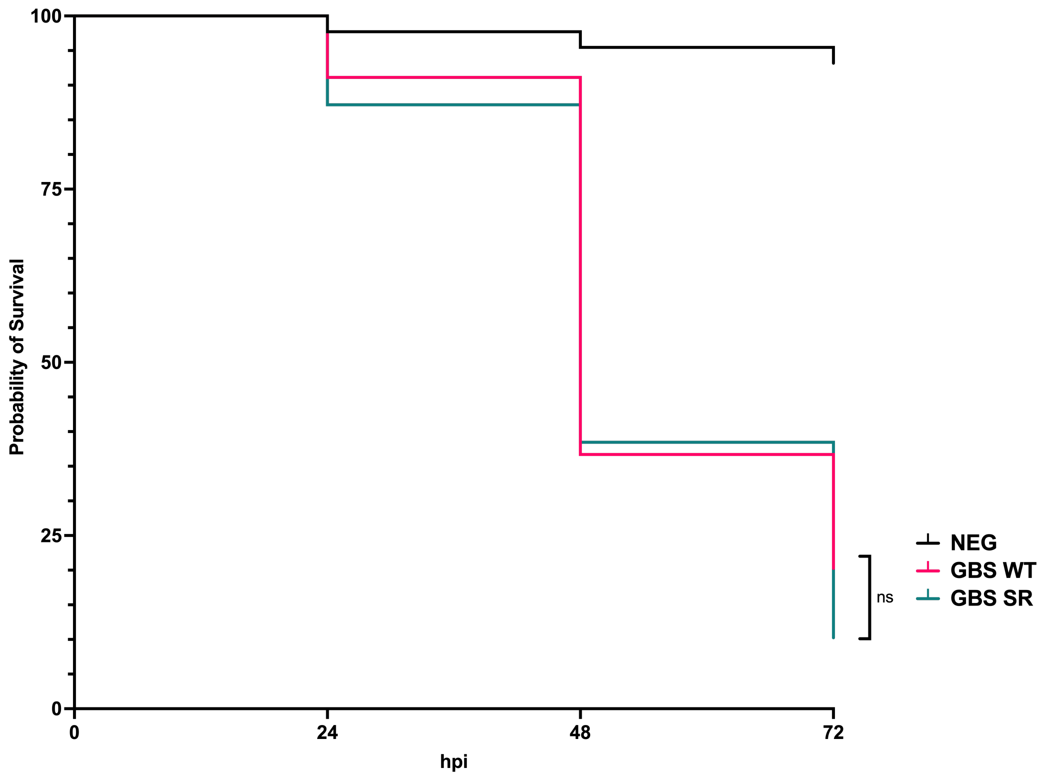

Supplemental Figure 9: Spontaneous antibiotic resistance to streptomycin does not affect virulence of GBS 515 Solo systemic infection was induced by injecting 2dpf larval zebrafish with either 40cfu of wild-type GBS 515 (GBS WT) or streptomycin resistant GBS 515 (GBS SR). Survival of zebrafish was monitored ever 24 hours for 72 hours total. Experiments were performed in triplicate and statistical significance of Kaplan-Meier survival curves were calculated using a log rank (Mantel-Cox) test. *$p<0.05$.

Supplemental figure legends

Supplemental Figure 1: GBS and *C. albicans* growth was not altered in co-cultures compared to solo cultures of the pathogens following 24hr culture in nutrient rich (THY) media GBS and C. albicans were incubated in nutrient rich media in solo and in co-cultures and viable colony counts were calculated following 24 hours of incubation **A.** GBS growth in solo and co-cultures with *C. albicans* following 24 hours of incubation **B.** *C. albicans* growth in solo and co-cultures with GBS following 24 hours of incubation. Experiments were performed in triplicate and statistical significance was calculated using an unpaired two-tailed student's t-test *p<0.05.

Supplemental Figure 2: GBS increase in growth when co-cultured with C. albicans is not serotype dependent. Colony growth of GBS COH1 (serotype III) following solo or co-culture with *C. albicans* at 24 hours post culture in nutrient poor media. Experiment was repeated 4 times and statistical significance was calculated using an unpaired two-tailed student's t-test *p<0.05.

Supplemental Figure 3. The ability of *C. albicans* to enhance the growth of GBS in co-cultures appears to be strain dependent. A. Colony growth of GBS following solo or co-culture with three different clinical strains of *C. albicans* at 24 hours in nutrient poor media. B. Colony growth of *C. albicans* growth following solo or co-culture with GBS at 24 hours post culture in nutrient poor media. was not significantly different in the presence of GBS for any of the *C. albicans* strains. *C. albicans* strains: VVC indicates a strain from a vulvovaginal candidiasis patient, and COL indicates a strain from an asymptomatic individual (46,47). 1887,

strain SP-01887; 4314, strain SP-14314; 6884, strain SP-16884. Results are from combined experiments, and experiments were performed in triplicate. Statistical significance was calculated by unpaired two tailed student t-test, *$p<0.05$, **$p<0.005$.

Supplemental Figure 4: GBS strain GBS 515 does not alter the ability of *C. albicans* to form hyphal filaments in RPMI media. The ability of *C. albicans* to form hyphal filaments in solo or co-cultures with GBS strain GBS 515 was quantified by taking images of solo and co-cultures using confocal microscopy. The percentage of area covered by hyphal filaments in the field of view was calculated using ImageJ (94,95). Above results are combined experiments from 3 separate replicates, and statistical significance was quantified using an unpaired two-tailed students t-test.

Supplemental Figure 5: The presence of GBS does not significantly change the overall growth of *C. albicans* in nutrient poor media. Growth of *C. albicans* strains CAF2-FR and $NRG1^{OEX}$-iRFP 24 hours post culture in nutrient poor media in solo *C. albicans* cultures or co-cultures with GBS 515. Experiments were performed in triplicate and statistical significance was calculated using unpaired two-tailed student's t-test *$p<0.05$.

Supplementary Figure 6. Hyphal formation plays a role in antibiotic susceptibility of GBS during co-cultures with *C. albicans.* Both CAF2-FR (WT) and $NRG1^{OEX}$-*iRFP* (yeast-locked) *C. albicans* strains increase growth of GBS during co-cultures. Erythromycin (A) and clindamycin (B) appear to be less effective against GBS when GBS is also cultured with *C. albicans* that is able to form hyphal filaments (CAF2-FR). While the amount of GBS recovered

following antibiotic treatment was also enhanced in the presence of Yeast-Locked *C. albicans* (*NRG1^{OEX}-iRFP*) it was not as efficient at reducing the efficacy of ERM against GBS as the hyphal *C. albicans* strain, indicating that the presence of hyphal filaments is beneficial to GBS in its ability to resist antibiotic treatment.

Supplemental Figure 7: Hyphal formation is not necessary for protection of GBS from the antibiotic clindamycin by *C. albicans* in yolk-sac infections Solo or co-infections of GBS and *C. albicans* using a yolk sac injection method on 2dpf zebrafish larvae. For solo infections zebrafish were injected with either 20 cfu of GBS 515 or 20 cfu of *C. albicans* yeast-locked strain NRG1^{OEX}-iRFP . For co-infections zebrafish were injected with 10 cfu of GBS and 10 cfu of *C. albicans* (totaling 20 cfu) together. The negative control was 1 nL of 5% PVP in PBS. Experiments were replicated 3 times with 15-20 fish per experimental condition. Graph shows pooled date of 3 experimental replicates **A.** Zebrafish survival of systemic solo and co-infection of GBS and *C. albicans* untreated or treated with 6μg/mL dose of clindamycin in the tank water of the fish at the time of initial infection. **B.** Tables describing zebrafish survival and infection group comparisons where differences in survival percentage were found to be statistically significant for systemic solo and co-infections of GBS and *C. albicans* untreated or treated with 6μg/mL clindamycin Statistical significance of Kaplan-Meier survival curves were calculated using a log rank (Mantel-Cox) test. ****$p<0.0001$.

Supplemental Figure 8: **Systemic co-infection with *C. albicans* does not significantly increase GBS burden *in vivo* in untreated infection or infection treated with the antibiotic clindamycin.** The bacterial and fungal burden in infected zebrafish was calculated following

yolk sac injection of solo or co-infections of GBS and *C. albicans* into 2dpf larval zebrafish. Solo injections had either 20 cfu of GBS 515 or 20 cfu of *C. albicans*. Co-infections had 10 cfu of GBS and 10 cfu of *C. albicans* (totaling 20 cfu) together. **A.** The average GBS bacterial burden in zebrafish 24 hours following untreated or clindamycin treated systemic solo or co-infection with *C. albicans*. **B.** The average *C. albicans* fungal burden of zebrafish 24 hours following untreated, or clindamycin treated solo or co-infection with GBS. Experiments were performed 4 times, with 8 fish homogenized and plated per infection replicate (n=256 fish). Above results are from combined experiments, with statistical significance calculated using one-way ANOVA with Tukey's correction *$p<0.005$.

Supplemental Figure 9: Spontaneous antibiotic resistance to streptomycin does not affect virulence of GBS 515 Solo systemic infection was induced by injecting 2dpf larval zebrafish with either 40cfu of wild-type GBS 515 (GBS WT) or streptomycin resistant GBS 515 (GBS SR). Survival of zebrafish was monitored ever 24 hours for 72 hours total. Experiments were performed in triplicate and statistical significance of Kaplan-Meier survival curves were calculated using a log rank (Mantel-Cox) test. *$p<0.05$.

Methods for Supplemental Figure 4:

To analyze the area of the field of view covered by hyphal filaments in images taken using confocal microscopy, solo cultures of *C. albicans* strain CAF2-FR and co-cultures of GBS strain GBS 515 GFP and CAF2-FR were cultured together for 24 hours with shaking at 37°C in RPMI media as previously described in this manuscript. To image CAF2-FR solo or co-cultures with GBS 515 GFP following 24 hours of growth, 7 μL of each culture were added to a glass slide

and covered with a 22 x 22 mm cover slip. After the cultures were mounted on the glass slides confocal images were taken using an Olympus IX-81 inverted microscope containing a FV-1000 laser scanning confocal system (Olympus, Waltham, MA). The fluorescent proteins EGFP (488nm/505 to 525 nm excitation/emission) and Far-Red (635nm/ 655 to 755 nm excitation/emission) were detected using laser/optical filters with a 20X objective. Single-slice images were taken and were processed using FluoView (Olympus, Waltham, MA). To quantify the percentage of area covered by hyphal filaments in solo or co-cultures of CAF2-FR and GBS 515 WT a modified version of a open source ImageJ protocol was used (1, 2). First, a calibration slide image was used to set the measurement parameters in ImageJ. Following the setting of the measurement parameters, images of solo and co-cultures were analyzed by first separating the channels and using the red channel for analysis to remove background noise caused by GBS 515 GFP. The separation of channels converted the image to an 8-bit image. Following this, hyphal filaments were carefully traced at a pixel thickness of 3 pixels for all images to avoid skewed results due to differences in fluorescence for hyphal filaments. Following the tracing, background noise was removed from the image using the "Subtract Background" feature in ImageJ. Then, images were converted to binary images using the "Make Binary" tool in ImageJ. Following the conversion to a binary image, the analyzing tool "Analyze particles" was used, which provided the total area covered by hyphal filaments in the field of view, as well as the percentage of area covered by filaments in the field of view. 15 images per culture type were analyzed, and 3 replicates were performed, with a total of 45 images per solo or co-culture being analyzed for statistical analysis. Statistical analysis was performed by comparing the average percentage of area covered by hyphal filaments in solo *C. albicans* cultures to co-cultures with GBS using an unpaired student's t-test using GraphPad Prism.

Supplemental References

1.Reinking L. 2007. Examples of Image Analysis Using ImageJ. ImageJ.net. Retrieved 9 August 2024.

2.Reinking L. 2007. Examples of Image Analysis Using ImageJ. ImageJ.net. https://imagej.net/ij/docs/pdfs/examples.pdf. Retrieved 9 August 2024.
